# Physical activity and sarcopenic obesity: definition, assessment, prevalence and mechanism

**DOI:** 10.4155/fsoa-2016-0028

**Published:** 2016-07-14

**Authors:** Duck-chul Lee, Robin P Shook, Clemens Drenowatz, Steven N Blair

**Affiliations:** 1 Department of Kinesiology, College of Human Sciences, Iowa State University, Ames, IA 50011, USA; 2 Department of Exercise Science, University of South Carolina, Columbia, SC 29208, USA; 3 Department of Epidemiology & Biostatistics, University of South Carolina, Columbia, SC 29208, USA

**Keywords:** exercise, handgrip strength, muscle mass, physical activity, physical fitness, physical function, sarcopenia, sarcopenic obesity, walking speed

## Abstract

Sarcopenic obesity is the coexistance of sarcopenia and obesity. Modern sarcopenia definition includes low muscle mass, weak muscle strength (handgrip strength) and poor physical function (slow walking), although the clinical definition of each varies worldwide. The cut-points for low muscle mass for men and women using appendicular lean mass divided by height (kg/m^2^) are ≤7.0 and ≤5.4 in Asians, and ≤7.23 and ≤5.67 in Caucasians, respectively. The cut-points for weak handgrip strength (kg) for men and women are <26 and <18 in Asians, and <30 and <20 in Caucasians, respectively. The cut-point for slow walking is ≤0.8 m/s in men and women. Current data suggest the potential benefits of physical activity and fitness on sarcopenic obesity in older adults.

## Lay abstract

Sarcopenic obesity (SO) is the combination of low muscle mass (sarcopenia) and obesity. The average prevalence of SO is about 5–10%. Older adults with SO have higher risks of mobility disability, cardiometabolic diseases and mortality. The medical problems related to SO are much greater than that in sarcopenia or obesity alone. Current studies suggest the potential benefits of physical activity, fitness and resistance exercise on the prevention and treatment of SO in older adults. This review highlights the importance of the development of effective public health strategies to prevent, delay and treat SO in older people.

**Figure 1.**
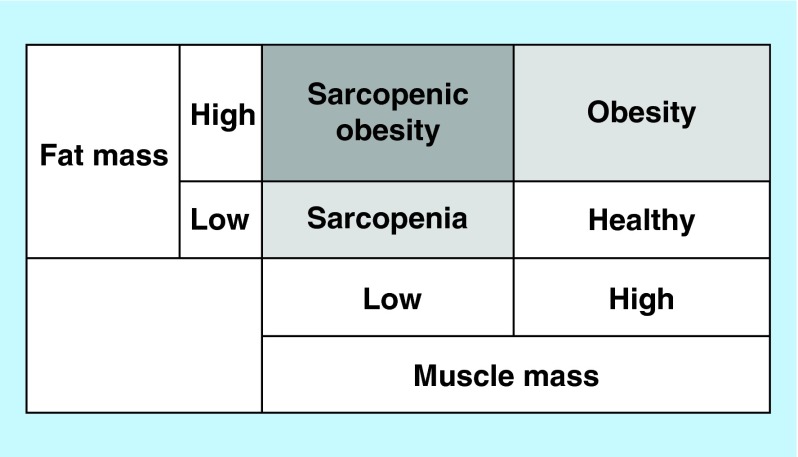
**Sarcopenic obesity by body composition phenotype.**

**Figure 2.**
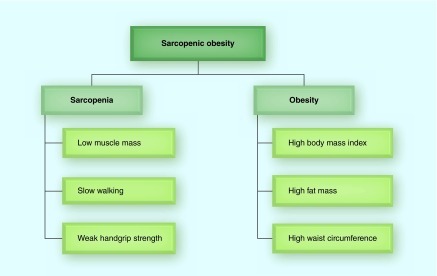
**Diagnostic criteria for sarcopenic obesity.**

**Figure 3.**
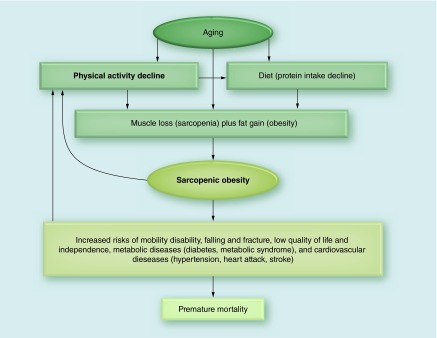
**Relationship between physical activity and sarcopenic obesity.**

Sarcopenia from the Greek ‘sarco’ for flesh and ‘penia’ for loss originally referred to the loss of muscle mass as a natural aging process [1]. In addition, older adults tend to gain fat mass with increasing age with a potential of developing obesity. Based on the national survey data published in 2014, more than one-third (35%) of American older adults are obese [2]. Additional concern is the rapid growing of the elderly population in most developed countries [3], resulting in a potent epidemiological confluence of risk factors for numerous health-related conditions. Considering that body composition includes both muscle and fat mass, there are four different body composition phenotypes, as described in Figure 1. The combination of high muscle mass and low fat mass is generally considered a healthy combination. Low muscle mass refers to sarcopenia and high fat mass refers to obesity. From a clinical perspective, the most concerning is the combination of low muscle mass plus high fat mass, termed sarcopenic obesity (SO) due to the coexistence of sarcopenia and obesity. SO is more common in older adults than young adults due to the natural changes in body composition associated aging.

Compelling evidence has shown that older adults with SO have higher risks of mobility disability [4–6], metabolic diseases [7,8], hypertension [9], cardiovascular diseases [10,11] and mortality [12–14], which is also related to significantly higher healthcare costs [15]. These clinical problems related to SO are much greater than that in sarcopenia or obesity alone [4,9,12,13], which suggests that sarcopenia and obesity have independent and additive adverse effects on health in the elderly. However, despite this significant and rising public health concern, there is very little evidence on SO. Therefore, investigating and developing effective SO prevention and treatment programs should be a priority based on the dramatically increasing health impact of SO in aging populations.

Although a certain degree of muscle loss and fat gain due to aging is inevitable, the good news is that SO is a modifiable condition, thus could be prevented and treated following effective therapy. Among related factors to SO, physical activity has been recognized as a key lifestyle factor to prevent and delay muscle loss and obesity with aging [16–18]. However, a few data are currently available on the effects of physical activity on SO in older adults, although there is accumulating evidence suggesting health benefits of physical activity on either sarcopenia or obesity. One of the major reasons of the limited data on SO is due to the differences in the definition and assessment of sarcopenia and SO among various working groups on sarcopenia. Also, data on the possible mechanisms of SO in various health conditions and the role of physical activity on the development of SO are still lacking. Therefore, the current review focuses specifically on SO including its clinical definition, assessment, diagnostic criteria, prevalence and potential mechanisms. We also explored and summarized the associations of physical activity, physical fitness and exercise training with SO in older adults.

## Method

In this review on physical activity and SO, we used PubMed to identify relevant peer-reviewed journal articles (the last search was conducted on 18 December 2015). The search keywords included ‘physical activity’ or ‘exercise’ or ‘fitness’ and ‘sarcopenic obesity’ using the advanced search method. We included all original human studies from cross-sectional surveys, observational cohorts and clinical trials that are not limited by publication year, population age, gender or country. However, conference abstracts, review articles, editorials or commentaries were not included. A total of 96 articles were initially identified. We read each abstract and further reviewed references from selected and eligible articles for additional research. To be included in the quantitative analyses, we required data on risk ratios (odds ratios or relative risk) in observational studies or changes in SO variables in intervention studies, comparing active or exercise groups against inactive or control groups. We found a total of six original research articles that investigated the associations of physical activity, fitness or exercise with SO; five cross-sectional studies and one randomized controlled trial. In addition to study design, the characteristics of the selected studies were further assessed by study population, measurement of physical activity, fitness or exercise program, definitions of sarcopenia and obesity and the prevalence of SO. The data extracted from the selected studies are presented in Table 1 for comparisons among studies.

## Assessment & definition of sarcopenic obesity

As mentioned previously, there currently is no universally adopted definition of SO, primarily due to the variations in the definition of sarcopenia (Table 2). Modern definitions of sarcopenia as a progressive medical condition includes not only loss of muscle mass, but also weakness of muscle strength (handgrip strength) and/or poor physical function (performance), which are well-established health predictors. Handgrip strength is a strong predictor of all-cause and cardiovascular mortality in people of diverse economic and sociocultural backgrounds based on a large longitudinal population study conducted in 17 countries [25]. The common physical function test, gait speed, is a significant predictor of disability [26] and survival [27]. In addition, there is supporting evidence indicating stronger associations of muscle strength with mobility limitations in older adults [28], but weak or no associations of muscle mass alone with mortality [29]. These data support the concept that consideration should be made of all three sarcopenia criteria: muscle mass, muscle strength and physical function.

### Assessment of sarcopenia

#### Muscle mass

In recent studies, body composition to measure muscle mass was most commonly acquired from whole body dual-energy x-ray absorptiometry (DXA). For skeletal muscle mass (SMM), appendicular lean mass (ALM) is generally used, which is calculated as the sum of lean mass in arms and legs without including fat and bone mass using DXA. ALM is also referred to as appendicular SMM or appendicular fat free mass. To define sarcopenia, relative muscle mass is typically utilized because absolute muscle mass is highly correlated with height or weight. Similar to body mass index calculation (total body weight divided by height squared, kg/m^2^), most sarcopenia definitions utilized the ALM index, ALM in kg divided by height squared in meter (kg/m^2^) [30–32] or divided by body mass index (BMI) [33], as a ratio of muscle mass to height or BMI. The cut-point of the height adjusted ALM index for low muscle mass was mostly established as two SD below the mean value of sex-specific reference values in healthy young adults (18–40 years), which was originally proposed by Baumgartner *et al*. [36]. One of the limitations of using ALM from DXA is that ALM does not include trunk muscles such as chest and back muscles, one of the largest muscle groups in humans. It is because the trunk has body organs (heart, lung and intestines) and DXA cannot accurately distinguish these from muscle mass. However, because people use their arms and legs in most daily activities as well as during resistance exercise to develop chest and back muscles, it is likely that trunk muscle mass is highly correlated to ALM. Therefore, using ALM from DXA is reasonable, although using total body lean mass including trunk muscles is ideal in future studies with more advanced technologies for total body composition assessment [37]. Also, based on its accuracy and availability, DXA is useful and practical for research and clinical use. Further, DXA has relatively low radiation compared with other imaging technologies such as computed tomography (CT) or MRI, which are more precise, yet expensive and have more radiation exposure. However, all these imaging technologies and equipment (DXA, CT and MRI) are not portable, which is important in large epidemiological studies in populations. Therefore, more research is needed to define sarcopenia and SO using other traditional or modern technologies that are safe, inexpensive and widely available including anthropometry (e.g., midupper arm or calf circumference) [38,39], bioelectrical impedance analysis (BIA) [40] and ultrasound [37].

#### Muscle strength

Handgrip (grip) strength has been widely used for muscle strength measurement, because it is inexpensive, easy to use and well correlated with most relevant health outcomes such as mortality [25]. Handgrip strength is measured by a handheld dynamometer, mostly using Jamar dynamometer and the maximum value from either hand or combined from both hands are analyzed [30]. However, because leg strength is more related to physical functions such as gait, chair standing and stair climbing, leg strength tests also have been utilized, especially in research studies. Common leg strength tests are knee flexion and extension at various velocities using isokinetic equipment (e.g., Biodex). This isokinetic test measures muscle power (work per unit time), which is the capacity of muscles to rapidly exert force as a measure of the explosiveness of the muscle. Measuring power may also be important in sarcopenia research in older adults because it is suggested that power could be a better predictor of functional capacities, as power is lost faster than strength during aging [41,42]. Another strength test is the one repetition maximum (1-RM) test, commonly using bench and/or leg presses. This test is traditionally used by athletic or fitness trainers to evaluate training programs. However, there is also strong evidence indicating health benefits of increased total body muscle strength measured by 1-RM test in general populations [43–45]. Nevertheless, because isokinetic and 1-RM tests need special equipment and proper training, they are less practical in clinical use. Therefore, more feasible as well as reliable lower body and total body strength tests, which are highly correlated with physical function, should be continuously developed and validated.

#### Physical function

There are a wide range of physical function tests, including usual gait speed, 6 min or 400-m walk tests, timed get-up-and-go test, chair stand test and the short physical performance battery, which is a composite measure of balance, gait and leg strength. However, usual gait speed is the most popular physical function and performance test in clinical practice and sarcopenia research because it is simple, fast and easy to measure as a predictor of mobility limitations and mortality in general populations, as well as patients after cardiac surgery [46–48]. Habitual gait speed was mostly measured at the usual pace on a 4- or 6-m course, and the average or the best value was used. Inability to rise from a chair was also considered as an alternate definition of mobility disability [30,31].

### Definitions & prevalence of sarcopenia

Because sarcopenia no longer refers purely to loss of muscle mass, there are differences in how sarcopenia is defined. Table 2 shows various clinical definitions of sarcopenia by major professional organizations and groups on sarcopenia.

The European Working Group on Sarcopenia in Older People (EWGSOP) recommends using the presence of low muscle mass plus either low muscle strength or poor physical function for the diagnosis of sarcopenia [30]. They noted that defining sarcopenia using only muscle mass is too narrow and may be of limited clinical value based on the fact that muscle strength does not depend solely on muscle mass and the relationship between strength and mass is not linear [49]. They further defined conceptual stages as ‘presarcopenia’ (low muscle mass), ‘sarcopenia’ (low muscle mass plus either low muscle strength or poor physical function) and ‘severe sarcopenia’ (low muscle mass, low muscle strength and poor physical function) based on the single or combination of three sarcopenia criteria to help select appropriate treatment and recovery goals. To define sarcopenia, the EWGSOP recommends a cut-point at two SDs below the mean value of healthy young reference adults. To identify sarcopenia cases, they suggest initially measuring gait speed, as it is considered the easiest and most reliable assessment, using the cut-point value of ≤0.8 m/s (slow walkers). Then, assessments of either handgrip strength or muscle mass should be followed based on walking speed. For slow walkers, the muscle mass assessment is then completed to define sarcopenia. For fast walkers, the handgrip strength test is additionally suggested to define low muscle strength, then final muscle mass was assessed. Therefore, in both cases, low muscle mass in addition to poor physical function and/or poor handgrip strength is necessary to be diagnosed with sarcopenia. In terms of numeric definitions, the EWGSOP recommends several cut-points for muscle mass based on different studies and measurement techniques. Among those different cut-points for muscle mass, we selected ALM index measured using DXA of 7.23 kg/m^2^ for men and 5.67 kg/m^2^ for women, which is the same cut-point recommended by the International Working Group on Sarcopenia (IWGS) 1 year later [31]. These cut-points were also used in another study to compare different sarcopenia definitions [50]. Also, the EWGSOP provides two other cut-points for low total body SMM measured by BIA, including cut-points of <8.87 kg/m^2^ for men and <6.42 kg/m^2^ for women, which were validated by comparing with MRI [34]. The other provided BIA-based cut-points were <10.76 kg/m^2^ for men and <6.76 kg/m^2^ for women, which were developed based on a large US national sample of 4449 older adults [35].

In the sarcopenia definition by the IWGS, the diagnosis of sarcopenia was given to individuals with poor physical function plus low muscle mass, without considering handgrip strength [31]. Poor physical function is defined as individuals who are bedridden, nonambulatory, cannot independently rise from a chair or who have a measured gait speed <1.0 m/s (most easily identifiable measure). These individuals with poor physical function should be further examined for muscle mass. Low muscle mass is defined by ALM index of ≤7.23 kg/m^2^ in men and ≤5.67 kg/m^2^ in women using DXA. These cut-points of ALM index for sarcopenia were developed based on the sex-specific lowest 20% of the distribution of the ALM index from the Health ABC Study (3075 well-functioning men and women aged 70–79 recruited in 1997–1998 from a random sample of Medicare enrollees in Pennsylvania and Tennessee in USA) [51].

The Asian Working Group for Sarcopenia (AWGS) supports using both DXA and BIA to define low muscle mass for sarcopenia diagnosis [32]. They indicated that although DXA may be the most widely used method for muscle mass assessment in sarcopenia studies, BIA is suitable based on its portability, reasonable cost, fast processing, noninvasiveness, radiation-free functions and convenience, especially in community-based screening programs. They provide evidence suggesting a reliable estimation of ALM from BIA, using DXA as a reference method in community-dwelling Japanese older women [52]. In another study in elderly Taiwanese men, results of fat-free mass using BIA were also associated with the results using DXA [53]. The AWGS recommends using two SDs below the mean muscle mass of a young reference group or the lower quintile as the cut-point value. They also recommend using height-adjusted ALM index, and cut-points for low muscle mass as 7.0 kg/m^2^ in men and 5.4 kg/m^2^ in women by using DXA, and 7.0 kg/m^2^ in men and 5.7 kg/m^2^ in women by using BIA. However, the cut-points in Asians are lower than those of Caucasian populations using the same DXA, recommended by the EWGSOP and IWGS groups on sarcopenia. This is because of relatively low prevalence of sarcopenia in Asian studies, which is partly due to lower body weight in Asians, compared with Caucasians. There are also differences in socioeconomic factors, lifestyle and culture between Asians and Caucasians. Regarding muscle strength, most Asian sarcopenia research studies used handgrip strength, the AWGS also recommends it as a feasible and convenient measure, similar to other sarcopenia definitions. The cut-point values of low handgrip strength are suggested to be defined as 26 kg in men and 18 kg in women. Regarding physical function, the AWGS recommends using a 6-m usual walking speed. After extensive review of Asian data, slow walking speed was defined as ≤0.8 m/s, which is consistent with the EWGSOP and the US definitions below. However, they reported that there is a potential gender difference in the cut-point value of usual walking speed with a wide range from 0.6 to 1.2 m/s.

In the US Foundation for the National Institutes of Health Sarcopenia Project (FNIHSP), a total of 26,625 participants (11,427 men and 15,198 women) were included in the pooled data analyses from nine studies after excluding participants <65 years old [33]. This is one of the largest sarcopenia projects incorporating diverse populations from different races, ethnicities, geographic regions and a range of health and functional status. The average age was 75.2 years for men and 78.6 years for women. The average BMIs were 27.1 kg/m^2^ in men and 26.9 kg/m^2^ in women. They recommended sequential screening and case identification processes: screening for physical function first using gait speed, followed by strength assessment using handgrip strength and then assessment of lean mass using DXA. The FNIHSP selected a usual gait speed ≤0.8 m/s for poor physical function, which is the same cut-point suggested by the EWGSOP and the AWGS groups. They reported that 10% of men and 31% of women had gait speed ≤0.8 m/s. The final recommended cut-points for muscle weakness were handgrip strength <26 kg for men and <16 kg for women. Regarding muscle mass assessment, experts in the study preferred a measure that accounts for body mass index instead of height that was suggested earlier in other sarcopenia groups. The final recommended cut-points of ALM/BMI index for low muscle mass using DXA were <0.789 for men and <0.512 for women.

Based on a review of the recommendations by four major working groups on sarcopenia above, and a careful examination of the existing literature, we propose the following recommendations (Table 3).

First, slow walking (poor physical function) was most commonly defined as gait speed of ≤0.8 m/s in both men and women regardless of race and ethnicity. This cut-point is supported by the European, Asian and the US groups. For muscle strength and mass, it is more appropriate to use separate cut-points for Asians and Caucasians based on higher adiposity in Asians given the same body weight [54], lower handgrip strength in Asians [25], lower prevalence of sarcopenia in Asians [32] and different lifestyle and cultural factors between Asians and Caucasians of European origin. This approach is also in line with different cut-points for obesity between Asians (BMI ≥25 mg/m^2^) and Caucasians (BMI ≥30 mg/m^2^), suggested by the World Health Organization [55]. The recommended cut-points for low handgrip strength (muscle weakness) are <26 kg in men and <18 kg in women in Asians, as suggested by the Asian group and <30 kg in men and <20 kg in women in Caucasians, as suggested by the European group. However, the US group suggested lower cut-points (<26 kg in men and <16 kg in women) for low handgrip strength than that by the European group. Based on the lower levels of handgrip strength in Asians [25], using the higher cut-points for weak handgrip strength in Caucasians suggested by the European group would be reasonable. However, more studies are needed to identify universal cut-points for low handgrip strength in Caucasians, which should be predictable of health outcomes such as functional disability and mortality. Regarding low muscle mass to define sarcopenia, we recommend cut-points of ALM/height^2^ ≤7.0 kg/m^2^ in men and ≤5.4 kg/m^2^ in women using DXA in Asians, as suggested by the Asian group. For Caucasians, ALM/height^2^ of ≤7.23 kg/m^2^ in men and ≤5.67 kg/m^2^ in women using DXA were suggested by European and International groups. However, BMI adjusted ALM cut-points of <0.789 in men and <0.512 in women could also be used in Caucasians, as suggested by the US group. Also, in large population studies when DXA is not available, BIA-based cut-points for low muscle mass shown in Table 2 could be a practical option. All four major working groups on sarcopenia recommend low muscle mass as a core diagnostic criteria for sarcopenia. However, there is an inconsistency regarding other diagnostic criteria for sarcopenia. The European and Asian groups recommend that either slow walking speed or low handgrip strength should additionally be included for sarcopenia diagnosis, whereas the US group suggests to include both slow walking speed and low handgrip strength in addition to low muscle mass. On the other hand, the IWGS includes only slow walking speed, but not muscle strength, in their diagnostic criteria for sarcopenia in addition to low muscle mass. Therefore, more studies are needed whether using all three criteria or which two criteria increases diagnostic power. At this point, we recommend to include at least either slow walking speed or low handgrip strength in addition to low muscle mass based on the current recommendations by the European and Asian groups.

A recent study compared sarcopenia prevalence using different definitions based on data from nine studies in an older adults population aged ≥65 years old (a total of 7113 men and 2950 women were included in the analyses) [50]. They found that the prevalence of sarcopenia was higher in women than men. They also found lower prevalence of sarcopenia with the US definition (1.3% in men and 2.3% in women), compared with the European definition (5.3% in men and 13.3% in women) and Asian definition (5.1% in men and 11.8% in women). Therefore, BMI adjusted ALM compared with height adjusted ALM in defining sarcopenia may result in a more conservative definition. These different definitions, even in the same Caucasian populations, make it difficult to compare results between studies. Therefore, it is important to develop a universal criterion and definition for the diagnosis of sarcopenia.

### Definition & prevalence of sarcopenic obesity

Theoretically, SO has been narrowly defined by low muscle mass and high fat mass, as described earlier in Figure 1. However, recent SO studies have used an expanded diagnostic criteria for identifying both sarcopenia and obesity, which also incorporates single or combination of different assessments of sarcopenia and the quantification of both systemic and central adiposity (Figure 2).

In addition, the cut-point values of each sarcopenia and obesity criterion are also different between studies, depending on population, gender, age, race and ethnicity. Most studies used ALM index divided by height^2^ <2.0 SDs [4,5,19,56] or ALM index divided by weight ≤2.0 SDs [8,20,21,57] using DXA to define sarcopenia. However, other studies used the lowest two quintiles of SMM divided by height^2^ using BIA [22,23], handgrip strength lowest tertile [58,59] or walking speed ≤0.8 m/s [60]. To define obesity, some studies used BMI ≥30 kg/m^2^ in men and women [51,59] or BMI >27.5 kg/m^2^ in men [60], and others used different % body fat values of >27% in men and >38% in women [4], >28% in men and >35% in women [19], >30% in men and >40% in women [5,61] or the highest two quintiles of % body fat [22,23]. Instead of BMI or % body fat, some studies also used visceral fat area >100 cm^2^ by CT scan [8], or waist circumference upper tertile [58], ≥90 cm in men and ≥85 cm in women [20,21,57] or >102 cm in men and >88 cm in women [56]. Due to these various cut-point values of sarcopenia and obesity, comparing findings between studies are challenging. Also, the prevalence of SO varies significantly from 0 to 25% in older adults between studies depending on study populations and the definitions of SO. However, considering all studies together found above, the approximate average prevalence of SO in older adults is about 5–10%, and it is similar between men and women. In general, the SO prevalence is lower (3–8%) using height adjusted ALM index [4,5,60,61] compared with weight or BMI adjusted ALM index (6–10%) [20,21,56,57] in defining sarcopenia, as indicated earlier in the prevalence of sarcopenia [16,33,50]. Also, the SO prevalence is significantly higher in people aged ≥80 years, compared with that in older adults aged <80 years. However, SO prevalence was higher (16–25%) when a more arbitrary definition of SO was used such as the lowest two quintiles of muscle mass or the highest two quintiles of % body fat [22,23].

### Physical activity & sarcopenic obesity

Regular physical activity, including both aerobic and resistance exercise, is a significant modifiable factor for the prevention and treatment of obesity in the general population or sarcopenia in older adults. Although physical activity has generally been shown to prevent weight gain and reduce fat mass in people with obesity, while also improving muscle mass and strength in sarcopenic older people, there are still very limited data regarding the benefit of physical activity in individuals with SO. Table 1 shows the summary of current studies that have investigated the associations of physical activity, physical fitness or exercise training with SO in older adults.

In the Quebec Longitudinal Study involving 904 older adults (mean age: 74 years), the authors investigated the associations between SO and physical fitness using objective measures of body composition and physical fitness in independent older adults [19]. In both men and women, they found that both sarcopenic obese and nonsarcopenic obese groups had lower physical fitness levels (measured by timed up and go, chair stand, walking speed and leg stand), compared with nonsarcopenic normal weight individuals after adjusting for age, physical activity and the sum of medical conditions such as cardiac problems, stroke, diabetes, lung diseases, digestive diseases, arthritis and osteoporosis. However, the sarcopenic obese group had similar fitness levels compared with the nonsarcopenic obese group. Therefore, obesity rather than sarcopenia appears to contribute more to lower physical fitness in these well-functioning older men and women.

In the Korean study of 2221 elderly population (mean age: 70 years), investigators examined the associations of exercise and walking with SO [20]. Although all results did not reach statistical significance, they reported that men who participated in resistance exercise ≥3 times/week, flexibility exercise ≥3-times/week or walking ≥1 h/day had 53, 30 and 49% lower odds of SO, respectively, compared with no resistance exercise, no flexibility exercise or walking <30 min/day, respectively, after adjusting for age. Similar but slightly weaker associations were found in women. In addition, high serum insulin was significantly associated with increased risk of SO, whereas high vitamin D level was associated with lower risk of SO among metabolic and nutritional factors. In another Korean study, investigators used a nationally representative noninstitutionalized elderly sample of 2264 older adults aged ≥65 years (mean age: 78 years) to examine the association between physical activity and SO. They found that men participating in moderate (e.g., ≥600 MET-min/week) and high (e.g., ≥3000 MET-min/week) physical activity had 51 and 75% significantly lower odds of SO, respectively, compared with low activity that did not meet the moderate or high activity criteria [21]. These results were adjusted for age, education level, lifestyle factors (smoking and alcohol) and medical conditions (heart disease, stroke, diabetes, hypertension and hyperlipidemia). In women, only high, but not moderate, physical activity was associated with 57% lower odds of SO, compared with low activity in the same analysis model. Therefore, this study suggested that there is a gender difference in the relationship between physical activity and SO, with stronger associations in men than in women. However, these differences may be due to different physical activity patterns between men and women, rather than biologically driven. For example, men tend to engage in more vigorous-intensity sporting activity and resistance exercise, whereas women perform more light-intensity domestic activities.

In the Spanish study of 306 octogenarians (mean age: 83 years), physical fitness predicted the risk of SO in both men and women [22]. Specifically, leg and arm strengths, agility, walking speed and balance in men, and agility and balance in women were more strongly associated with SO. Another larger Spanish study in 2747 noninstitutionalized elderly population (mean age: 72 years) reported similar findings indicating that lower physical fitness levels were associated with an increased risk of SO [23]. Further, balance, aerobic capacity and walking speed were identified as the most sensitive fitness tests associated with the risk of SO with 70–80% significantly lower odds of SO in the highest tertile compared with the lowest tertile of each fitness test score in each gender after adjusting for age. However, body strength and flexibility were less related to the risk of SO with 20–70% lower odds of SO in the highest tertile compared with the lowest tertile of each fitness test score in both men and women.

We found only one randomized controlled trial that investigated the effects of a 15-week resistance training on physical function in 17 US older adults aged 60–90 years (mean age: 71 years) with SO at baseline [24]. Physical function was assessed using the short physical performance battery test including 4-m usual gait speed, chair stand and standing balance tests. Participants were randomly assigned either traditional strength/hypertrophy (SH) training or high-speed circuit (HSC) power training. The traditional SH training group performed three sets of 10–12 repetitions using 70% of their one repetition maximum on 11 exercises with 1–2 min of recovery between sets. The participants were instructed to perform the concentric and eccentric phases of each exercise in 2 s. The HSC power training group performed the same sets and repetitions using relatively lower intensity (lower weight) with no recovery between sets. Also, the participants were instructed to perform the concentric phases of each exercise as fast as possible, and perform the eccentric phase in 2 s. Investigators found a significant 20% improvement in physical function in the HSC power training, and a nonsignificant 7% improvement in the traditional SH training group. However, they found significant improvements in muscle strength and power using leg and chest press tests in both resistance training groups. This study clearly suggests the treatment effect of resistance exercise in SO patients, specifically that high-speed muscle power training (moving resistance at higher velocities) could be more beneficial to improve physical function. However, due to the small sample size (n = 17) and a short intervention (15 weeks), additional research on this topic is warranted.

All six studies suggested potential health benefits of physical activity, fitness and resistance exercise training on SO in older adults in different populations, although the strength of the associations were different between studies. Therefore, the results between studies must be interpreted with caution, and there are several factors affecting the differences among studies such as different methods to measure physical activity, fitness or body composition; different cut-point values to categorize individuals with sarcopenia and/or obesity; age and health conditions of the study populations and different study design. Five out of six studies used a cross-sectional study design, and there is only one small exercise intervention study with a short training period of 15 weeks. Therefore, causality between physical activity and SO cannot be established in most studies, and the effect of changes in physical activity over time on SO remains to be determined. Thus, longitudinal studies are needed to investigate long-term effects of physical activity and fitness on SO in older individuals. Also, more studies are needed to establish the most effective type or combination of exercise and its optimal amount and intensity for the prevention and management of SO. Furthermore, to minimize unfavorable adverse events, exercise prescription should consider the higher risk of injury or complications in the elderly due to clinical conditions such as osteoporosis or cardiometabolic diseases.

In addition to physical activity, diet including adequate protein intake and amino acid supplementation is another key factor in effectively enhancing body composition, as recommended for the management of sarcopenia and SO [17,18,62,63]. In a recent study, exercise combined with amino acid supplementation was most effective in improving muscle mass, strength and walking speed in women with sarcopenia [64]. Another strategy besides exercise and diet could include a pharmaceutical approach such as hormone therapy (growth hormone), since low levels of growth hormone are a risk factor for sarcopenia [65]. SO is a continuous process for years and decades, although it progresses and accelerates faster after the age of 60. Therefore, multiple lifelong approaches combining physical activity, diet and potentially pharmaceutical interventions should be considered for the prevention and treatment of SO.

## Mechanisms linking physical activity to sarcopenic obesity

SO is a multifaceted medical condition with a complicated etiology and results in varying health consequences. The central cause of SO in older adults is the age-related decline in muscle mass and accumulation of fat mass, directly or indirectly through changes in physical activity and diet, as illustrated in Figure 3. SO, however, can also occur in young adults. For example, due to repetitive extreme diet for weight loss (yo-yo diet) following excessive caloric restriction and unbalanced diet, which may cause muscle loss, weight regain and obesity [66]. However, fat distribution may be different in SO in older adults with more intramuscular (fat infiltration into muscle) [28,67] and visceral fat increase and subcutaneous fat decrease with aging [68].

Possible reasons for the decline in aerobic and muscle-strengthening physical activity by aging include a decreased occupational activity after retirement, osteoarthritis and muscular skeletal injuries, fear of falling and fracture risk due to osteoporosis and decreased interest and physical ability in sports and exercise. In addition, decreased appetite and gastrointestinal function by aging may also reduce protein intake and digestion, which attributes to muscle loss due to decreased muscle protein synthesis [69], leading to sarcopenia and SO.

SO is associated with or leads to several adverse outcomes with increased risks of mobility disability [4–6], falling and fracture [70], low quality of life and independence [49], metabolic diseases [7,8] and cardiovascular diseases [9–11], which increases risk of premature mortality [12–14]. Potential mechanisms for the development of SO and its health problems include increased insulin resistance [20,56], increased chronic inflammation [58,71], decreased hormones (testosterone, growth hormone, DHEA, IFG-1) [72], decreased neuromuscular function [73–75] and decreases in energy expenditure and fat oxidation [76].

SO and chronic diseases may also cause physical inactivity due to reduced exercise capacity (decreased cardiorespiratory fitness and muscular strength), physical limitations [77] and increased fatigue after exercise. Therefore, breaking this vicious cycle by increasing physical activity and sufficient protein intake should be emphasized to prevent SO and its related health consequences.

## Discussion (current limitations & future direction)

Current sarcopenia and SO definitions have been primarily developed based on data available from cross-sectional analyses, and there is still no universal definition with consensus. This lack of unified definition for SO has contributed inconsistent findings on the prevalence of SO and the associations between SO and health outcomes. Therefore, it is important to continue to discuss and develop consensus criteria for a clinical definition of SO by comparing different definitions in various populations including those with increased risk of functional disability, bone fracture, joint replacement surgery and other musculoskeletal injuries. It is also important to measure changes in SO variables over time in the same individuals. Therefore, longitudinal studies and randomized controlled trials should be performed to investigate the causation and prognosis of SO. These studies can contribute to finding the effects of changes in ALM, muscle strength, gait speed and fat mass on SO. For clinical trials, including the primary SO parameters (muscle mass, muscle strength and physical function, fat mass and waist circumference) as well as relevant clinical outcomes such as cardiometabolic markers, chronic inflammatory markers, quality of life, falling and fracture, activities of daily living and independence (hospitalization) should be considered to provide evidence based SO prevention and treatment strategies.

Based on the significant differences in body composition and muscle strength between men and women, sarcopenia definitions for muscle mass and strength were presented separately by gender. However, regarding gait speed, the same cut-point value is used to define poor physical function, although the value is different between men and women as presented in USA [33] and the Asian groups [32] on sarcopenia. Therefore, further study may be warranted addressing the question whether different cut-points of gait speed for men and women should be utilized in accordance to other measures of physical function. Regarding muscle strength, the current recommendation is to use unadjusted handgrip strength because it is simple to use clinically, although handgrip strength is highly correlated with body weight. The FNIHSP study also reported that BMI adjusted ALM index had consistent associations with mobility limitations between different study samples in women [78]. However, continuous clarification and further investigation is needed if body weight or BMI adjusted handgrip strength or muscle strength should be used based on its relations to various health outcomes, as used in other studies on muscle strength and health outcomes [43–45]. In addition, while gait speed and handgrip strength are easy to measure, more practical and comprehensive assessment of functional ability and total body strength such as the sitting-rising test [79] should be developed. Those new tests should be related to daily activities and health outcomes such as quality of life and mortality in older adults, and could be easily measured by individuals at home without using instruments.

In defining and diagnosing sarcopenia, the current cut-point values for muscle mass were mostly developed based on the lowest 20% or two SDs below the mean value of young healthy reference group from a specific study population. This method is originally designed to parallel the definition of osteoporosis (reduced bone mass), which has been associated with risk of fracture. However, low muscle mass alone is not consistently associated with relevant adverse health outcomes. Therefore, more studies are urgently needed to have reliable and validated reference values for low muscle mass in general populations around the world, as suggested by the FNIHSP [33]. In addition, the cut-point values should preferably be developed based on prospective outcome studies, instead of those simple statistical methods (the lowest 20% or two SDs below the mean value of the reference group).

BMI is easy to measure and commonly used to define obesity worldwide as a representative measure of whole body obesity in population levels. However, because BMI does not consider body composition such as muscle and fat mass, its accuracy in research is continuously debated, especially in older adults and specifically in the assessment of SO. Compared with young adults, older adults could have more fat mass given the same BMI levels due to muscle loss by aging. Therefore, it is possible that older adults even in the normal weight category by BMI could have more fat mass, which increases the risk of cardiometabolic diseases such as diabetes or heart attack. Future studies on SO should measure body composition instead of BMI to define SO in older adults. Additionally, because obesity is simply defined by BMI without considering metabolic health and fitness, there are controversial issues regarding different obesity phenotypes such as metabolically healthy obesity [80], and obesity with high cardiorespiratory fitness levels [81,82] that may not be associated with increased risks of cardiometabolic diseases and mortality. There is also an obesity paradox indicating that obesity has some mortality benefits in individuals with heart diseases or Type II diabetes [83,84]. Also, a recent review reported mortality benefits in overweight and obesity in older adults [85,86], which is another obesity paradox in older adults. The answers to these controversial issues on different obesity phenotypes and paradox may be related to muscle mass and strength, especially in older adults. Therefore, future studies on obesity and SO should consider more comprehensive approaches including body composition and muscle mass in the analyses.

Another type of muscle disorder that should be studied is dynapenia, which is more closely related to muscle quality rather than muscle quantity (muscle mass). Possible causes of dynapenia include impairments in neural activation, decreased muscle contractile quality and reduced motor unit recruitment [74], which leads to significant loss of muscle strength. Loss of muscle mass due to sarcopenia also affects dynapenia. Several studies indicated that older adults with dynapenia have increased risk of functional disability [87], falling [70] and metabolic diseases [88], even after adjusting for sarcopenia [89]. In our earlier study, we found that individuals with lower muscle strength (the lowest 20th percentile) compared with the moderate or high muscle strength (upper 80th percentile) had over two-fold higher risk of developing metabolic syndrome in both young (<50 years) and old (≥50 years) men after adjusting for age, smoking and alcohol intake [90]. This association remained significant, although reduced, even after further adjustment for BMI and cardiorespiratory fitness in both young and old men. We also found that individuals with higher muscle strength had lower risk of developing obesity [44] and reduced all-cause, CVD and cancer mortality regardless of their body fatness [45,91]. Further, even hypertensive men with higher muscle strength (upper third) had 34% lower risk of all-cause mortality compared with lower muscle strength (lower third), after adjusting for body fatness and cardiorespiratory fitness [43]. These findings clearly support the importance of muscle strength as an independent risk factor for chronic diseases and mortality not only in older but also in general populations. However, there are still relatively limited data on muscle strength and resistance exercise on health outcomes compared with cardiorespiratory fitness and aerobic exercise on health. Therefore, more studies are required on this important topic of resistance exercise and muscle strength in relation to dynapenia, sarcopenia and SO.

There are also several important questions that should be considered in relation to physical activity and SO. What type and amount of physical activity and exercise are most effective for SO prevention and treatment in older adults with and without functional limitations? What are the practical strategies to promote and increase habitual physical activity in older adults? How can physical activity be combined with other lifestyle factors including sufficient protein intake, healthy diet and chronic disease management for prevention and treatment of SO?

## Conclusion & future perspective

Based on the current review, the average prevalence of SO in older adults ranges from 5 to 10% depending on its definitions and study populations, and the prevalence is significantly higher in people aged ≥80 years. The number of older adults aged ≥60 years is expected to more than double, from 841 million in 2013 to over 2 billion in 2050 globally [3]. Accordingly, the percentage of older adults is expected to increase from 11.7% in 2013 to 21.1% by 2050. This older population is also aging themselves, and the proportion of the oldest old people aged ≥80 years is projected to increase dramatically. Further, older adults are projected to exceed the number of children for the first time in 2047 worldwide [3]. Based on the above estimation of the prevalence of SO and older adult population, SO may affect 40–80 million people today and will affect 100–200 million in the next 35 years globally.

Sarcopenia and SO studies have emerged only in the past 2–3 decades, and research in this area is still in its infancy. However, the population with SO around the world has major health, social and economic consequences, often with limited coverage of social security system in many developing countries [3]. Therefore, most countries are expected to experience increasing challenges to deal with the significant impact of SO regarding mobility disability, hospitalization, increased chronic diseases, mortality and healthcare cost.

Although we found many important questions that remain unanswered, it is important to note that SO can be prevented, delayed and treated by maintaining or adopting a healthy lifestyle, including increasing aerobic and muscle-strengthening activities and sufficient protein intake and healthy diet. Specifically, we found potential health benefits of physical activity, fitness and resistance exercise training on SO in older adults in different populations in this review (Table 1). However, most findings are based on cross-sectional studies. Therefore, longitudinal studies are clearly needed to investigate long-term effects of physical activity and fitness on SO in older individuals. We hope that the current review guides researchers and health professionals around the world to further refine definition, diagnosis and classification of sarcopenia and SO for both research studies and clinical practice. It is also important to develop simple and easy measurement techniques and tools that are reliable and valid, thus it can be included in the routine geriatric assessment to provide targeted and appropriate intervention. Simultaneously, we should also continue to identify effective public health strategies and programs to combat SO for health promotion and quality of life improvement for millions of older people around the world.

**Table 1.** **The associations of physical activity, physical fitness or exercise training with sarcopenic obesity.**

**Study (year), country**	**Sample size**	**Mean age (range)**	**Study design**	**Physical activity/fitness/exercise**	**Sarcopenia definition**	**Obesity definition**	**Sarcopenic obesity prevalence**	**Main results**	**Ref.**
Bouchard *et al*. (2009), Canada	904 (465 women)	74 (68–82)	Cross-sectional	Physical fitness using four tests (timed up and go, chair stand, walking speed and leg stand)	Two SDs below the mean ALM/height^2^ of young adults (20–35 years) using DXA (<8.51 kg/m^2^ in men and <6.29 kg/m^2^ in women)	% body fat of ≥28% in men and ≥35% in women using DXA	Men: – Nonsarcopenic and nonobese: 30% – Nonsarcopenic and obese: 31% – Sarcopenic and nonobese: 20% – Sarcopenic and obese: 19% Women: – Nonsarcopenic and nonobese: 23% – Nonsarcopenic and obese: 59% – Sarcopenic and nonobese: 7% – Sarcopenic and obese: 11%	Physical fitness was more strongly associated with obesity than sarcopenia	[19]
Hwang *et al*. (2012), South Korea	2221 (1257 women)	70 (≥60)	Cross-sectional	Self-reported physical activity on frequencies of resistance exercise, flexibility exercise and walking time per day	Two SDs below the mean (ALM/weight) × 100 of healthy young adults (20–39 years) using DXA (29.53% in men and 23.20% in women)	Waist circumference ≥90 cm in men and ≥85 cm in women	Men: – Nonsarcopenic and nonobese: 70% – Sarcopenic and obese: 6% Women: – Nonsarcopenic and nonobese: 57% – Sarcopenic and obese: 7% ‘Nonsarcopenic and obese’ and ‘Sarcopenic and nonobese’ were excluded in the analyses	Although not significant, higher levels of physical activity were associated with lower risk of SO	[20]
Ryu *et al*. (2013), South Korea	2264 (1324 women)	78 (≥65)	Cross-sectional	Physical activity using the International Physical Activity Questionnaire	Two SDs below the mean (ALM/weight) × 100 of healthy young adults (20–39 years) using DXA (cut-points not reported)	Waist circumference ≥90 cm in men and ≥85 cm in women	Men: – Nonsarcopenic: 89% – Sarcopenic: 11% – Sarcopenic and obese: 7% Women: – Nonsarcopenic: 88% – Sarcopenic: 12% – Sarcopenic and obese: 7%	Higher levels of physical activity was associated with a reduced risk of SO	[21]
Muñoz-Arribas *et al*. (2013), Spain	306 (230 women)	82.5	Cross-sectional	Physical fitness using eight tests (leg stand, chair stand, arm curl, chair sit-and-reach, back scratch, 8-foot up-and-go, 30-m brisk walk and 6-min walk)	The lowest two quintiles of SMM/height^2^ Using BIA (<8.62 kg/m^2^ in men and <6.20 kg/m^2^ in women)	The highest two quintiles of% body fat using BIA (≥30.34% in men and ≥40.91% in women)	Men: – Nonsarcopenic and nonobese: 30% – Nonsarcopenic and obese: 15% – Sarcopenic and nonobese: 30% – Sarcopenic and obese: 25% Women: – Nonsarcopenic and nonobese: 30% – Nonsarcopenic and obese: 17% – Sarcopenic and nonobese: 28% – Sarcopenic and obese: 25%	Adequate levels of physical fitness were associated with a lower risk of SO	[22]
Pedrero-Chamizo *et al*. (2015), Spain	2747 (2102 women)	72 (65–92)	Cross-sectional	Physical fitness using eight tests (leg stand, chair stand, arm curl, chair sit-and-reach, back scratch, 8-foot up-and-go, 30-m brisk walk and 6-min walk)	The lowest two quintiles of SMM/height^2^ using BIA (<8.62 kg/m^2^ in men and <6.20 kg/m^2^ in women)	The highest two quintiles of% body fat using BIA (≥30.34% in men and ≥40.91% in women)	Men: – Nonsarcopenic and nonobese: 36% – Nonsarcopenic and obese: 25% – Sarcopenic and nonobese: 23% – Sarcopenic and obese: 16% Women: – Nonsarcopenic and nonobese: 37% – Nonsarcopenic and obese: 23% – Sarcopenic and nonobese: 24% – Sarcopenic and obese: 16%	Higher levels of physical fitness were associated with a reduced risk of SO	[23]
Balachandran *et al*. (2014), USA	17 included in the analysis (16 women)	71 (60–90)	RCT	15-week traditional strength/hypertrophy training versus high-speed circuit training	Meeting ≥2 out of the three sarcopenia criteria: SMM/height^2^ using BIA (<10.76 kg/m^2^ in men and <6.76 kg/m^2^ in women), gait speed (<1 m/s) and handgrip strength (<30 kg in men and <20 in women) according to the EWGSOP definition	Body mass index >30 kg/m^2^	All 17 study participants had SO at baseline The primary outcome was the short physical performance battery (SPPB) test including 4-m usual gait speed, chair stand and standing balance tests Secondary outcomes include muscle strength and power tests using leg and chest presses	A significant 20% improvement in SPPB was found in the high-speed circuit training group. Both resistance training groups showed significant improvements in muscle strength and power	[24]

ALM: Appendicular lean mass (arms and legs); BIA: Bioelectrical impedance analysis; DXA: Dual energy x-ray absorptiometry; EWGSOP: European Working Group on Sarcopenia in Older People; RCT: Randomized controlled trial; SD: Standard deviation; SMM: Skeletal muscle mass (whole body); SO: Sarcopenic obesity.

**Table 2.** **Clinical definition of sarcopenia.**

**Group**	**Diagnostic criteria and cut-points for sarcopenia**					**Ref.**
	**Physical function**		**Muscle strength**		**Muscle mass**	
European Working Group on Sarcopenia in Older People (EWGSOP, 2010)	Gait speed ≤0.8 m/s	Or	Handgrip strength	And	DXA ALM/height^2^	[30]
			Men: <30 kg		Men: ≤7.23 kg/m^2^	
			Women: <20 kg		Women: ≤5.67 kg/m^2^	
					BIA SMM/height^2^	
					Men: <8.87 kg/m^2†^	
					Women: <6.42 kg/m^2†^	
					Men: <10.76 kg/m^2‡^	
					Women: <6.76 kg/m^2‡^	
International Working Group on Sarcopenia (IWGS, 2011)	Gait speed <1.0 m/s			And	DXA ALM/height^2^	[31]
					Men: ≤7.23 kg/m^2^	
					Women: ≤5.67 kg/m^2^	
Asian Working Group for Sarcopenia (AWGS, 2014)	Gait speed ≤0.8 m/s	Or	Handgrip strength	And	DXA ALM/height^2^	[32]
			Men: <26 kg		Men: ≤7.0 kg/m^2^	
			Women: <18 kg		Women: ≤5.4 kg/m^2^	
					BIA ALM/height^2^^§^	
					Men: ≤7.0 kg/m^2^	
					Women: ≤5.7 kg/m^2^	
Foundation for the NIH Sarcopenia Project (FNIHSP, 2014)	Gait speed ≤0.8 m/s	And	Handgrip strength	And	DXA ALM/BMI	[33]
			Men: <26 kg		Men: <0.789	
			Women: <16 kg		Women: <0.512	

^†^The cut-points were developed based on total body skeletal muscle mass using BIA in Asians [34].

^‡^The cut-points were developed based on total body skeletal muscle mass using BIA in Caucasians [35].

^§^The cut-points were developed based on appendicular skeletal muscle mass using BIA in Asians [32].

ALM: Appendicular lean mass (arms and legs); BIA: Bioelectrical impedance analysis; BMI: Body mass index; DXA: Dual energy x-ray absorptiometry; SMM: Skeletal muscle mass (whole body).

**Table 3.** **Recommendation for the diagnostic criteria and cut-points for sarcopenia.**

**Population**	**Physical function (gait speed)**		**Muscle strength (handgrip strength)**		**Muscle mass (DXA ALM/height^2^)**
Asian	Men: ≤0.8 m/s	Or	Men: <26 kg	And	Men: ≤7.00 kg/m^2^
	Women: ≤0.8 m/s		Women: <18 kg		Women: ≤5.40 kg/m^2^
Caucasian	Men: ≤0.8 m/s	Or	Men: <30 kg	And	Men: ≤7.23 kg/m^2^
	Women: ≤0.8 m/s		Women: <20 kg		Women: ≤5.67 kg/m^2^

ALM: Appendicular lean mass; DXA: Dual energy x-ray absorptiometry.

**Executive summary**

Sarcopenic obesity (SO) is the combination of low muscle mass plus high fat mass based on the body composition phenotypes. However, modern definition of sarcopenia includes low muscle mass, low muscle strength (weak handgrip strength) and poor physical function (slow walking).
Based on the sarcopenia definitions suggested by different working groups, the cut-points for low muscle mass for men and women using appendicular lean mass divided by height^2^ are ≤7.0 kg/m^2^ and ≤5.4 kg/m^2^ in Asians, and ≤7.23 kg/m^2^ and ≤5.67 kg/m^2^ in Caucasians, respectively. The recommended cut-points for weak handgrip strength for men and women are <26 kg and <18 kg in Asians, and <30 kg and <20 kg in Caucasians, respectively. The most common cut-points for slow walking is ≤0.8 m/s in both men and women regardless of race and ethnicity. However, there is a wide range of variations in the definition, assessment and diagnosis of SO for both studies.
Although the prevalence of SO varies significantly from 0 to 25% in older adults between studies depending on study populations and the definitions of SO, the average prevalence of SO is about 5–10%, which is similar between men and women. The prevalence of SO is significantly higher in people aged ≥80 years.
Older adults with SO have higher risks of mobility disability, metabolic diseases, hypertension, cardiovascular diseases and mortality, which is also related to significantly higher healthcare cost. These clinical problems related to SO is much greater than that in sarcopenia or obesity alone.
Despite the limited data, current studies suggest that there are potential health benefits of physical activity, fitness and exercise training on the prevention and treatment of SO in older adults. Sufficient diet such as adequate protein intake and amino acid supplementation is another key factor for the management of SO.
Potential mechanisms on the development of SO and its health problems include increased insulin resistance, increased chronic inflammation, decreased hormones, decreased neuromuscular function and decreased energy expenditure and fat oxidation due to aging.
Given the rapidly increasing health impact of SO in aging populations in most developed countries, the current review highlights the importance of the urgent investigation and development of universal definition, assessment and diagnosis of SO for both research studies and clinical practice. In addition, we should continue to identify effective public health strategies and programs to prevent, delay and treat SO for millions of older people around the world.
